# On simulated annealing phase transitions in phylogeny reconstruction

**DOI:** 10.1016/j.ympev.2016.05.001

**Published:** 2016-08

**Authors:** Maximilian A.R. Strobl, Daniel Barker

**Affiliations:** a School of Biology, University of St Andrews, St Andrews, Fife KY16 9TH, UK; b School of Mathematics and Statistics, Mathematical Institute, North Haugh, St Andrews, Fife KY16 9SS, UK

**Keywords:** Phylogeny, Optimisation, Heuristic methods, Simulated annealing, Phase transition, Search landscape

## Abstract

Phylogeny reconstruction with global criteria is NP-complete or NP-hard, hence in general requires a heuristic search. We investigate the powerful, physically inspired, general-purpose heuristic simulated annealing, applied to phylogeny reconstruction. Simulated annealing mimics the physical process of annealing, where a liquid is gently cooled to form a crystal. During the search, periods of elevated specific heat occur, analogous to physical phase transitions. These simulated annealing phase transitions play a crucial role in the outcome of the search. Nevertheless, they have received comparably little attention, for phylogeny or other optimisation problems. We analyse simulated annealing phase transitions during searches for the optimal phylogenetic tree for 34 real-world multiple alignments. In the same way in which melting temperatures differ between materials, we observe distinct specific heat profiles for each input file. We propose this reflects differences in the search landscape and can serve as a measure for problem difficulty and for suitability of the algorithm’s parameters. We discuss application in algorithmic optimisation and as a diagnostic to assess parameterisation before computationally costly, large phylogeny reconstructions are launched. Whilst the focus here lies on phylogeny reconstruction under maximum parsimony, it is plausible that our results are more widely applicable to optimisation procedures in science and industry.

## Highlights

• Comprehensive study of the simulated annealing heuristic search in phylogeny.
• Investigation of specific heat and phase transitions for 34 real world multiple alignments.
• Each multiple alignment produces a unique specific heat profile.
• Specific heat profiles will have diagnostic value for algorithmic optimisation.

## Graphical abstract

**Figure:**
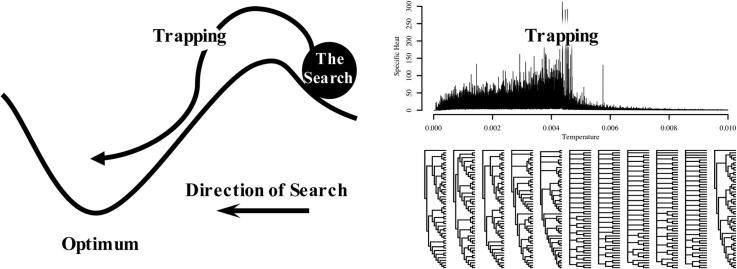

## 1 Introduction

Global optimisation is an important step in modern phylogeny reconstruction. To identify the most plausible phylogenetic tree under a given criterion, one has to find the tree with optimal fit amongst all conceivable tree topologies. The maximum parsimony criterion (MP) requires optimisation of tree length (Fitch, 1971) and maximum likelihood (ML), as the name suggests, requires optimisation of a likelihood function (Felsenstein, 1981). However, MP is an NP-Complete problem (Foulds and Graham, 1982) and ML phylogeny reconstruction is NP-Hard (Roch, 2006). It follows that in order to be sure of obtaining the optimal tree, one would theoretically need to examine all feasible topologies; a number which grows factorially with the number of taxonomic units in the analysis (Felsenstein, 1978a). Even for studies of moderate size, this number exceeds the number of atoms in the universe and makes an exhaustive search infeasible. Despite reductions in complexity achieved by the branch-and-bound algorithm (Hendy and Penny, 1982) and revolutions in computing power, it is likely that run time for exact optimisation will remain astronomical.

As a workaround, heuristic optimisation algorithms are used. Instead of setting out to determine all globally optimal solutions, these methods use shortcuts to aim to find optima approximately. As such, heuristics allow one to obtain very good solutions in practical time scales when exact optimisation is too costly. This is also true in phylogeny reconstruction. Heuristic searches underlie the majority of today’s MP and ML phylogeny reconstruction programs, for example PAUP, TNT, PhyML and RAxML (Swofford, 2003, Goloboff et al., 2008, Guindon et al., 2010, Stamatakis, 2014). However, in emplyoing heuristic searches we fundamentally lose the guarantee of global optimality. Using a heuristic search it remains possible that even if the true phylogeny was hypothetically inferable from an alignment, one might not be able to retrieve it – simply because the algorithm did not happen to search a specific area of the search space. This problem is particularly pressing for analyses involving many taxonomic units.

The increasing ease and decreasing costs of DNA sequencing allow opportunities for very large phylogeny reconstructions. At the same time, phylogeny is being applied to even more areas of the life sciences. Applications include, for example, ancestral state reconstruction (Lutzoni et al., 2001), assessing biodiversity (Flynn, 2011), predicting gene function (Eisen, 1998, Barker et al., 2007) and investigations of cancer and pathogen evolution, with implications for treatment (Gerlinger et al., 2012, Köser et al., 2012). Such analyses build on the solution of large and complex optimisation problems, making further development of heuristic searches in phylogeny increasingly urgent.

An important step towards more efficient algorithms is a better understanding of the nature of heuristic searches. In the current paper we present an analysis of the search behaviour of the simulated annealing heuristic. Simulated annealing, available in the phylogeny reconstruction packages LVB, MetaPIGA, SAMPARS and RAxML (Barker, 2004, Helaers and Milinkovitch, 2010, Richer et al., 2013, Stamatakis, 2014), is inspired by the physical processes occurring during the crystallisation of a liquid by gentle cooling (Kirkpatrick et al., 1983, Cerny, 1985). Convergence to the final solution is controlled by a parameter mimicking temperature in a physical system, with certain ranges of this parameter, called phase transitions, being particularly important to the search (Kirkpatrick et al., 1983, Cai and Ma, 2010, Hasegawa, 2012). In the following paper, we investigate the nature and role of these phase transitions during simulated annealing searches under the MP criterion. We identify the phase transitions for 34 real world phylogeny problems. We find that properties of phase transitions are repeatable for the same analysis and input and vary across different inputs, but in all cases correspond to the onset of effective resolution of the tree structure. Subsequently, we discuss how knowledge of the phase transition can help advance our understanding of the functioning of simulated annealing. We hypothesise phase transitions can serve as a useful diagnostic for finding suitable parameterisations for the search and be a stepping stone for future algorithmic improvements. Whilst in the current study we focus on phylogeny reconstruction under MP, conceptual links between MP and ML (see ‘Maximum Parsimony’, below) and the general nature of the simulated annealing algorithm make it plausible that our results are relevant for other areas of phylogeny reconstruction and beyond.

### 1.1 Maximum parsimony

For evolution of discrete traits on a given tree topology, the minimum number of changes consistent with the observed characters is known as tree length. Maximum parsimony seeks to find the topology of lowest length for the data matrix at hand.

Fitch (1971) provides a rapid, dynamic programming method to calculate the length for a given tree topology.

Because of its simplicity the most parsimonious tree problem provides a good model for studying global optimisation in phylogeny reconstruction and was therefore the chosen focus of this study. Calculations are significantly quicker than for the ML case and one avoids complications arising from needing to select appropriate models and parameters for the data at hand, which might confound the interpretation. This allows one to gain a fundamental understanding of the optimisation algorithm which in the future can then be extended to more complex optimality criteria.

Nevertheless, our immediate results are of general interest to the phylogeny community. Although MP is usually regarded as a distinct method, the MP tree is also the ML tree, at least under a constrained ‘no common mechanism’ model (Tuffley and Steel, 1997, Steel and Penny, 2000; see also Cavalli-Sforza and Edwards, 1967, pp. 239–240). For the model implied by MP the number of parameters increases with the amount of data, leading to statistical inconsistency (Felsenstein, 1978b, Yang, 2006). Simulation studies suggest the biological accuracy of MP is lower than that of statistically consistent ML approaches (e.g. Huelsenbeck, 1995). But even where one desires statistical consistency, the MP tree may still be useful as an initial tree, for further refinement by ML whose evaluation function is more time-consuming to calculate. This approach combines the speed of calculation of tree length (MP) for the initial part of the search, with a consistent approach to finding the final result (ML). This is an option in, for example, PhyML (Guindon et al., 2010).

Tree length varies with the input data, as well as with the optimality of the tree for those data. For greater comparability across different input files we used the tree consistency index (CI), with a theoretical range of 0–1 (Kluge and Farris, 1969), which seeks to normalise tree length and improve comparability. CI provides a measure of the amount of homoplasy on the proposed tree. For a given data matrix, a higher consistency index indicates reduced homoplasy, hence a shorter tree. Tree CI is given by:

(1) $$\text{CI}=\frac{K}{\text{Tree length}}\text{,}$$

where *K* is the sum of the minimum number of changes for each column in the multiple alignment individually (without reference to any tree structure). As such, *K* is a theoretical minimum of the number of mutations that has to have occurred to produce the given alignment from a single ancestor sequence. To cast the problem as a minimisation, which is more typical, we seek trees of minimum homoplasy index (HI), where HI = 1 – CI (Swofford, 1993).

### 1.2 Simulated annealing

The simulated annealing algorithm was independently developed by Kirkpatrick et al. (1983) and Cerny (1985). It is inspired by the processes which occur during the cooling of physical systems and is a simple but powerful optimisation technique. If a liquid is cooled slowly, the atoms anneal to form a crystal structure that minimises their energy. Since the particles in the liquid are in continuous motion at each instance they go through a plethora of positions and arrangements, some of which will be energetically more favourable than others. When cooling is applied and the system is given sufficient time at each temperature, the distribution of states visited will shift towards – and finally become – the very low energy crystalline state (van Laarhoven and Aarts, 1988).

Simulated annealing mimics this process: in the same way that the physical system seeks the state of minimal energy, it seeks the solution of minimal cost. The algorithm will start with an initial, often randomly generated solution *X* and perturb it according to some neighbourhood function *N*, to propose an updated solution *X*′. Similarly to how particles will always adopt a state that is an improvement over the current one, the algorithm will always move to *X*′ if it has lower cost. However, also if *X*′ has higher cost, in accordance with the physical analogy it will be occasionally accepted with probability:

(2) $$P_{\mathrm{acc}}=\exp(-\Delta H/T)\text{,}$$

where Δ*H* is the change in cost and *T* is a control parameter playing the role of temperature. The algorithm will perform a certain number of such moves to allow the system to equilibrate. Then the temperature *T* is decreased according to a decrement rule known as the cooling schedule. The cooling schedule is often chosen as a geometric law of the form: *T_n_*_+1_ = *α T_n_*, 0 < *α* < 1 (e.g. Barker, 2004, Kirkpatrick et al., 1983). If a certain number of temperature decrements fails to bring about further improvements, the system is considered frozen and the search is terminated. Despite its relative simplicity, simulated annealing yields high quality solutions for a wide range of optimisation problems (e.g. van Laarhoven and Aarts, 1988, Ingber, 1993, Lindorff-Larsen et al., 2005, Alavi and Gandomi, 2011, Kolish and Dahlmann, 2015), including phylogeny reconstruction (Lundy, 1985, Salter and Pearl, 2001, Barker, 2004, Stamatakis, 2005, Richer et al., 2013).

When a liquid is cooled, there is a distinct point at which the state of order of the system fundamentally changes and freezing and crystallisation begin. This phase transition from liquid to solid occurs when the specific heat of the system, a measure of the system’s ability to store heat, is at a peak. Taking the definition of the specific heat, *C*, as

(3) $$C=\frac{\sigma^{2}(\text{cost function})}{T^{2}},$$

where *σ*^2^ denotes the variance of the cost function and *T* is the current temperature, one can compute *C* for simulated annealing runs on the computer and observe similar transitions (Kirkpatrick et al., 1983, Dress and Krüger, 1987, Abramson et al., 1999). It appears that the peaks in the specific heat correspond to particularly important periods in the search (Kirkpatrick et al., 1983). Note that these phase transitions, during the simulated annealing search, are different from the phase transitions in time complexity known in computer science (Hasegawa, 2012).

In the original paper on the simulated annealing algorithm (Kirkpatrick et al., 1983), two peaks were reported in the specific heat, during runs on a chip placement problem. Subsequently, similar peaks have been reported for other optimisation problems, for example phylogeny reconstruction (Dress and Krüger, 1987) and timetabling (Abramson et al., 1999). Taking advantage of phase transitions, different algorithmic optimisation strategies have been proposed, such as reheating schemes (Abramson et al., 1999) or adaptive cooling schedules (Kirkpatrick et al., 1983). Nevertheless, phase transitions have received comparably little attention, usually being confined to side notes and figure legends, and the processes underlying their occurrence are yet to be fully understood. Little is known about whether the characteristics of the transition (e.g. the temperature at which they occur) or the number of transitions are specific to the particular problem case or to the class of optimisation problem. Clearly, such knowledge would improve our understanding of the simulated annealing search, with relevance for algorithmic optimisation.

In the first study of its kind we have recorded and analysed the specific heat during simulated annealing runs on a set of 34 instances of a particular optimisation problem – phylogeny reconstruction under MP. Utilising the hierarchical structure of phylogenies, we investigate the role of the transitions in the progress of the search and develop a more in depth understanding of the events occurring during the phase transition. We propose that study of the specific heat profile can serve as a diagnostic for choosing search parameters, allowing optimisation of simulated annealing-based phylogeny inference algorithms. Whilst our main focus lies on phylogeny, the general nature of the simulated annealing technique makes it likely that our results are also applicable more widely in science and industry.

## 2 Methods

### 2.1 Algorithm

We investigate the search dynamics of the simulated annealing algorithm in finding the most parsimonious tree. Experiments were conducted using a modification of LVB, version 3.1 (Barker, 2004; http://lvb.bio.ed.ac.uk).

LVB begins the search with a random topology and creates candidate solutions using nearest neighbour interchange and subtree pruning-regrafting alternately. LVB’s final hill-climbing search and storage feature for equally parsimonious trees were removed for our study to leave us with the canonical form of simulated annealing described in Section 1.2.

To obtain the specific heat *C* during a search we recorded the HI of the current ‘working’ solution at regular temperatures. At each temperature *T* the HI of the current working solution was stored every *l* = 10 moves*.* In total, *n* = 2000 moves were attempted before the temperature was decreased, to allow for equilibration to occur. The specific heat at temperature *T* can then be computed using Eq. (3), where we estimate *σ*^2^(cost function) from the sample of recorded HI values. To achieve comparable sampling of all temperature ranges we used a linear cooling schedule, which decreases the temperature by a constant *m* = 10^−7^. The values of *m, n* and *l* were chosen on the basis of preliminary experiments (not shown).

### 2.2 Phase transition study

We collected 46 nucleotide multiple alignments from the literature. These data matrices comprise all available, phylogenetically-oriented nucleotide multiple alignments associated with papers in *Systematic Biology* Volume 63, Issues 3 and 4. These include animal, viral and plant sequences and vary considerably in size, with 5–525 sequences and 402–58,950 nucleotide positions. Matrices were converted to PHYLIP format using Perl and BASH scripts and Mesquite (http://mesquiteproject.org). Sequence names were shortened to 10 characters and rendered unique within the alignment, where required, by incorporation of a three-digit code. Matrices after format conversion, and associated details, are given in Supplementary Data. Because of run times exceeding feasible limits, 12 matrices were excluded from the analysis.

For each of the 34 remaining alignments we performed 20 independent runs. Depending on the position of the peak, we started with an initial temperature of *T*_0_ = 2 × 10^–3^, *T*_0_ = 1 × 10^–2^ or *T*_0_ = 2 × 10^–2^. The initial temperature for each matrix is given in Supplementary Data (see Sections S1 and S2). We ensured that no two runs shared the same random number seed.

### 2.3 Detailed analysis

For nine multiple alignments, samples of candidate solutions were collected. At each temperature *n* = 2000 moves were attempted. Trees were obtained by writing out the current working solution every *l* = 100 moves at 20 linearly spaced temperatures. At each temperature sampled, consensus trees were created using the program consense in the PHYLIP package (Felsenstein, 1989) and figures were produced using Dendroscope (Huson et al., 2007). Trees were rooted using the outgroups specified in the original papers.

As discussed in Section 3.3, the minimum length *K* in Equation (1) acts as the Boltzmann constant in Equation (2). To evaluate the effect of changing *K* on the specific heat profiles, the protocol from Section 2.1 was repeated on 5 files with values of *K* equalling 0.1, 0.5, 2 and 10 times its original value, with 20 replicates per file.

### 2.4 Benchmarking

Five files of the 34 real-world multiple alignments were selected at random to serve as benchmarking cases. For each file the search was run 20 times from three different initial starting temperatures: *T*_1_ < *T*_2_ < *T*_3_, where *T*_1_ = 10^−4^, *T*_2_ = critical temperature and *T*_3_ = 2 *T*_2_ – *T*_1_. The critical temperature, *T*_2_, was determined as the mean of the temperatures at which the maximum specific heat had occurred during the experiments in Section 2.1. The number of proposed candidate solutions was held constant across all temperatures for each file. This constant was determined as the average number of proposed candidate solutions during 20 preliminary tests starting from *T*_3_. Equally parsimonious trees were not stored and LVB’s standard geometric cooling schedule was used, so as to give a canonical implementation as described in Section 1.2. The parameterisations for each file are given in Section S5.

All computational experiments were carried out on a computer cluster running Linux, with AMD Opteron 6320 2.8 GHz processors and between 128 Gb and 512 Gb DDR3-1600 ECC RAM per server. Data was analysed in R (http://www.R-project.org), with default parameters for boxplots.

## 3 Results and discussion

### 3.1 Phase transitions in phylogeny inference under MP

**Fig. 1:**
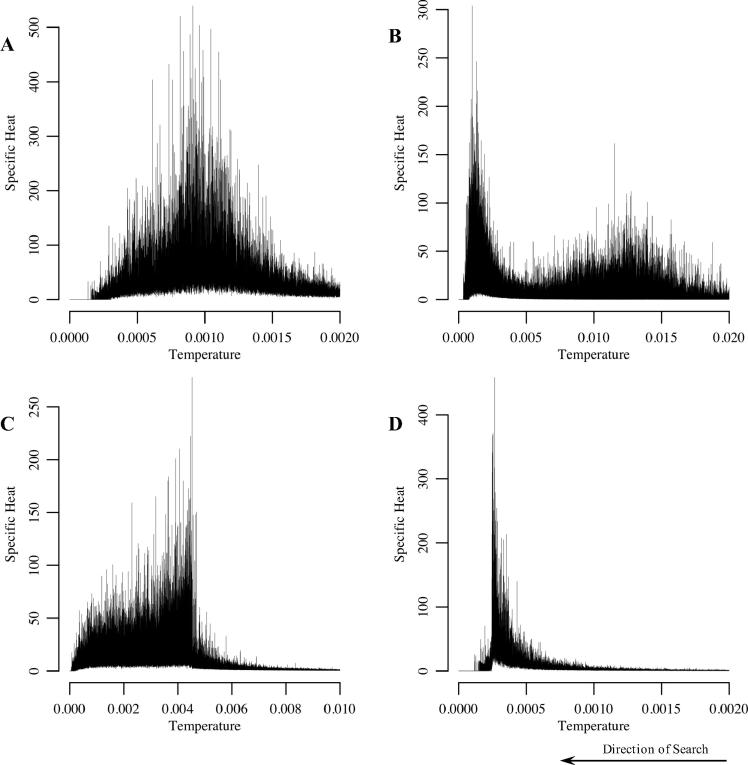
Examples of specific heat profiles observed. Each panel shows specific heat as a function of temperature for a single run. The search progresses right to left as the temperature is decreased. Whilst peak shape is preserved across different runs, with minor variations (see also Fig. 2), there is strong variation across input files, with each of the 34 alignments producing a unique profile. We observe symmetric single peaks (A – input file: ‘patient6’), double peaks (B – ‘VATI_ND2_Align_Final’), left-skewed (C – ‘Salicini1.smh’) and right-skewed single peaks (D – ‘Bahl’). For a full overview see Figs. S1–S3.

Phase transitions reflect the divide and conquer approach intrinsic to simulated annealing (Kirkpatrick et al., 1983). For phylogeny inference under MP, they were first noted by Dress and Krüger (1987). In all 34 cases we studied here, we also recorded the peaks in specific heat characterising phase transitions (Fig. 1). Moreover, whilst in 29 instances we observe single peaks, as in the single case-study presented by Dress and Krüger (1987), for five alignments we found two distinct maxima. To the best of our knowledge this is the first time that both single and double transitions have been reported for the same class of optimisation problem. Even greater numbers of peaks, as might have been expected from the multi-level hierarchical structure of phylogenies, were not observed.

**Fig. 2:**
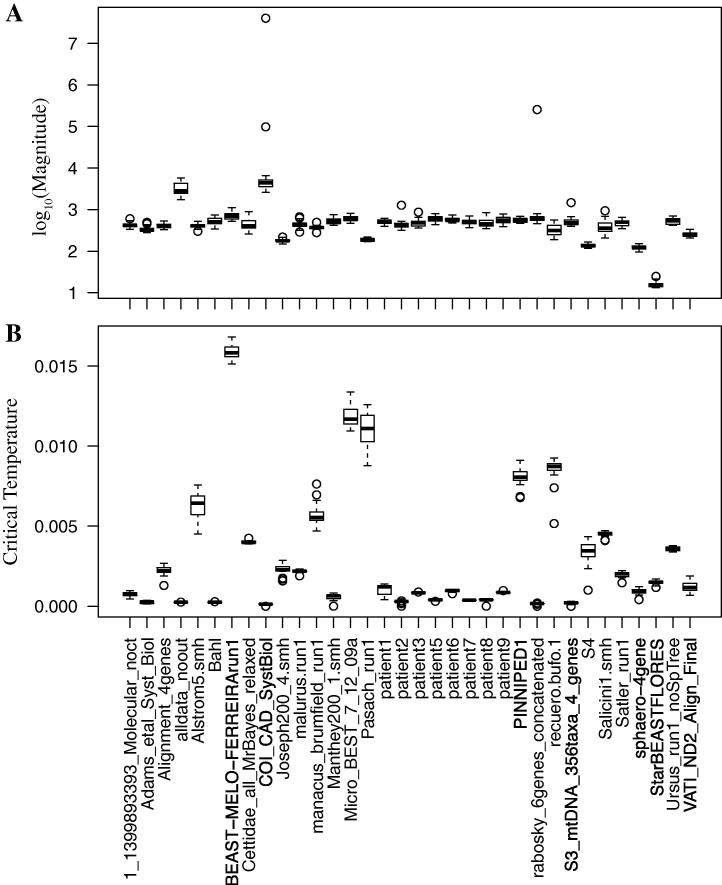
Boxplots of (A) log_10_(magnitude) and (B) critical temperature, for the 34 multiple alignments (using the largest peak where double transitions were observed). There is strong evidence of variation between alignments (Anova of log-transformed data: (A) *F*_33,646_ = 77.66, *p* < 2 × 10^–16^; (B) *F*_33,646_ = 86.60, *p* < 2 × 10^–16^).

Specific heat profiles differ strongly between multiple alignments. Whilst the characteristics of the specific heat curves are well preserved across the replicates for each individual alignment, we observe great variations in (i) magnitude of the maximum, (ii) temperature at which the maxima occur (‘critical temperature’) and (iii) peak ‘shape’ between cases. Each alignment corresponds to a unique profile (Fig. 1). We observe smooth bell-shaped curves with varying degrees of right and left skew, and profiles with sudden drops or rises as the temperature is decreased. Average magnitude of peaks differs by up to several orders of magnitude between different files and also critical temperature varies (Fig. 2). Furthermore, double transitions occur in diverse forms with some having a more pronounced high temperature maximum, whilst for others the lower temperature maximum is more visible. Moreover, separation of the two peaks varies greatly (for further discussion see Section S2).

Variation in the magnitude in specific heat can be explained by a greater number of sequences in the multiple alignment. Research into phase transitions in finite dimensional systems suggests an increase in the specific peak height as the size of the system increases (Challa et al., 1986). Indeed, we find positive correlation between the magnitude of the maximum specific heat and the number of sequences in the matrix (Pearson correlation, per-matrix means of log-transformed data; *r*^2^ = 0.51, d.f. = 31, *p* = 3 × 10^−6^). With more sequences in the alignment the number of possible solutions increases and so a greater range of states can be realised. Consequently, the variance in the cost function and so the specific heat increase. However, this does not account for the variation in critical temperature and number and shape of the peaks.

To the best of our knowledge, variation in the specific heat profile for different input files has not been reported before. In physical annealing, melting temperatures reflect the internal structure of materials. Analogously, we propose that simulated annealing phase transitions are related to characteristics of the optimisation problem – here, to characteristics of the particular multiple alignments. In the following we elaborate on this idea and show how certain properties of the problem and the algorithm manifest themselves in the specific heat profile, and discuss implications for algorithmic optimisation.

### 3.2 Differentiation by search space characteristics?

The simulated annealing search can be pictured as a walk in a mountainous landscape, where height represents cost and the space is the space of all possible trees. The aim of the walk is to find the deepest valley. The walk is initially random, since at high temperatures nearly all moves are accepted and the algorithm visits a range of valleys. As the temperature is decreased, the bias shifts towards downhill moves and the search is confined to smaller and smaller areas of the search space. Once the temperature has dropped below a certain point, the search becomes trapped in a single valley. The remainder of the time is spent exploring the valley and attempting to reach its lowest point (Schön, 1997, Salamon et al., 2002; Hasegawa, 2011).

**Fig. 3:**
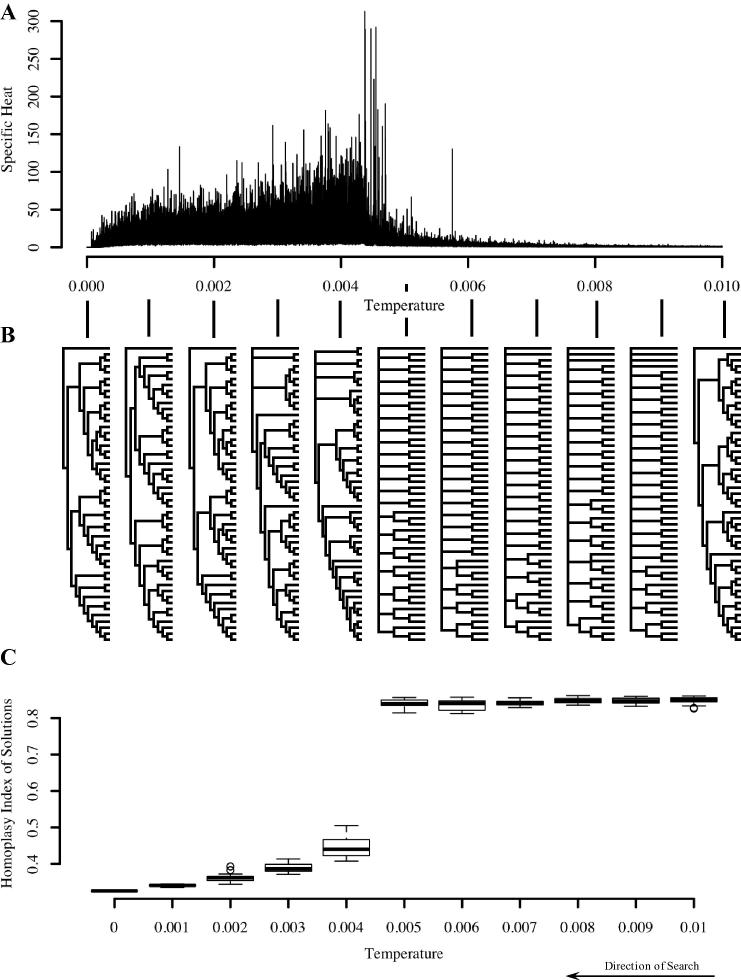
The period of the peak in specific heat corresponds to the trapping of the search in a particular neighbourhood. (A) Shows specific heat as a function of temperature for a single run on ‘Salicini1.smh’. (B) Shows a strict consensus tree for temperatures 9 × 10^−3^, 8 × 10^−3^, 7 × 10^−3^, 6 × 10^−3^, 5 × 10^−3^, 4 × 10^−3^, 3 × 10^−3^, 2 × 10^−3^, 1 × 10^−3^ created from samples of trees accepted at these temperatures. At *T* = 1 × 10^−3^ the initial tree and at *T* = 0 the final tree are shown. (C) Displays the homoplasy index of the sampled trees as a function of temperature for the same run. As the temperature falls below the critical temperature in (A), accepted trees become significantly more similar in structure (B), suggesting the search has become trapped in a particular region of the search space.

We observe that, for phylogeny inference problems, this trapping point appears to coincide with the critical temperature (Fig. 3). If we consider the solutions accepted by the algorithm at various temperatures, we find that, as the temperature is lowered below the critical temperature, the solutions accepted by the algorithm become very similar in structure. This is illustrated in Fig. 3B. The consensus tree reflects structure which is shared across all trees in the sample, hence a better-resolved consensus tree indicates greater similarity of the trees in the sample. As the temperature decreases below the critical temperature, the consensus trees transition from unresolved to resolved, indicating convergence to a family of similar tree topologies – that is, a particular region of the search space.

We hypothesise that the shape of the peak reflects the structure of the search landscape. The difference in the solutions considered by the algorithm before and after the transition is rather remarkable. Before the transition the average cost is high, after it is very low and close to the final cost (Fig. 3C). Rather than being merely ‘trapped’ by the neighbourhood of solutions, the search is ‘absorbed’. A plausible explanation for this might be the structure of the search space. For several combinatorial optimisation problems it has been reported that the number of solutions near the rim of a valley is exponentially greater than the number near the centre (Tayarani-Najaran and Prügel-Bennett, 2014). This means that until a certain temperature, when the search becomes sufficiently downhill oriented, the minima inside the valley are essentially invisible to the search as it is ‘distracted’ exploring the large number of solutions on the rim (Tayarani-Najaran and Prügel-Bennett, 2014). We suggest that a similar mechanism is at work here and that the rapid convergence we observe in Fig. 3 reflects such an exponential structure. For further discussion see also Section S3.

**Fig. 4:**
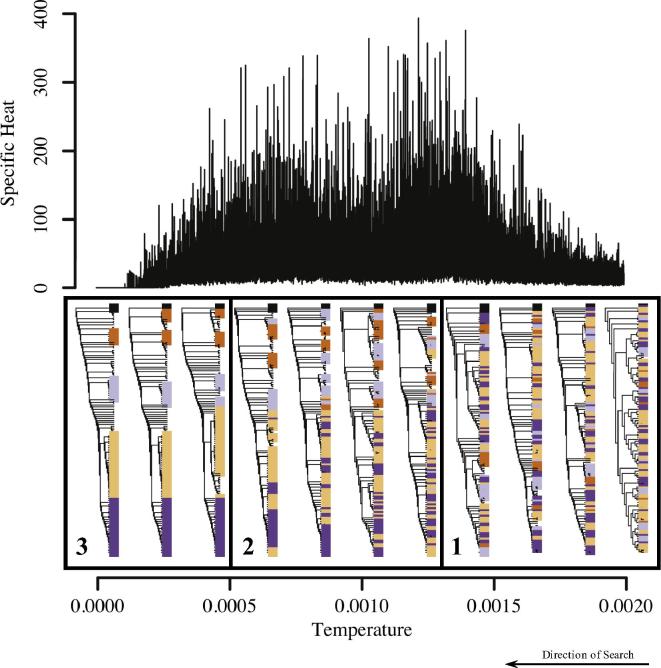
The resolution of the tree structure for an instance where we observe a double transition (‘patient1’). Trees have been selected at random from the set accepted by the search at temperatures 2 × 10^−4^, 4 × 10^−4^, 6 × 10^−4^, 8 × 10^−4^, 10 × 10^−4^, 12 × 10^4^, 14 × 10^−4^, 16 × 10^−4^ and 18 × 10^−4^ as indicated in the figure. The trees shown at *T* = 20 × 10^−4^ and *T* = 0 are the initial and final trees. Colouring corresponds to five clades in the final tree. As the temperature is decreased, first, taxa are arranged into coarse-grained clades with some incorrect placements (Phase 2), then membership of these clades is refined (Phase 3). Transition between the phases coincides with peaks in the specific heat, providing evidence that the gross-structure fine-structure paradigm proposed by Kirkpatrick et al. (1983) also holds in this case.

Moreover, we propose that accessibility of the deepest-lying regions is also reflected in the shape of the specific heat profile. Ease-of-access is determined by the number of minima and their relative influence (Sibani et al., 1993, Tayarani-Najaran and Prügel-Bennett, 2014). Each minimum acts on the direction of the search until the temperature is too low to allow further switching (Schön, 1997, Salamon et al., 2002, Fischer et al., 2011). If there is only a single minimum, descent is straightforward since there is only a single attracting point. However, if there are several neighbouring deep-lying minima (or potentially even terraces or plateaus of similarly optimal trees; Sanderson et al., 2011) then a large number of trees compete for attracting the search making progress difficult and slow. Such a system is called ‘frustrated’ (Vannimenus and Toulouse, 1977, Pearce and Turner, 2015) and we propose that the extent of frustration is reflected in the shape of the specific heat peaks. The more easily accessible the minima and the less the frustration, the more succinct the peak. For example, in Fig. 3(A), the peak is very succinct and the difference in the consensus trees once the critical temperature has been passed is stark. We conjecture that here the final minimum is easily identifiable. In contrast, detailed investigation of the double transitions reveals a different pattern of convergence (Fig. 4). Initially gross and then fine structure is resolved and progress is generally much slower. We hypothesise that double transitions reflect a hierarchical valley system with high frustration. Initially clusters of minima compete (big valleys corresponding to gross structure); later in the search, minima within a cluster compete (sub-valleys or plateaus corresponding to fine structure). The fact that we observe varying degrees of separation of the two peaks for different multiple alignments (e.g. ‘VATI_ND2_Align_Final’ and ‘S4’ in Section S2) plausibly reflects different degrees of frustration. For an attempt to explicitly map out the search space structure and relate it to the specific heat profile the interested reader is referred to Section S3.

It would be a useful future direction to explore the relationship further, and to investigate why three or more peaks were not observed. In addition to providing deeper insights into the dynamics of the simulated annealing search this could plausibly be used to assess the amount of frustration in a problem as an indication of its difficulty and conditioning. The problem of frustration is known to be a major challenge in phylogeny inference problems (Goloboff, 1999, Sanderson et al., 2011).

### 3.3 Visualising parameterisation

We have discussed how characteristics of the input file might be reflected in the specific heat profile. The parametersiation of the search also influences the observed peaks, as we will now illustrate.

A parameter which has important influence on how the simulated annealing algorithm moves around the search space is the Boltzmann constant (van Laarhoven and Aarts, 1988, Ingber, 1993). It parameterises the probability function controlling the acceptance of uphill moves (Eq. (2)) and regulates how directed the search is at a particular temperature (van Laarhoven and Aarts, 1988). In the optimisation of HI, the theoretical minimum length *K* plays the role of the Boltzmann constant since the acceptance probability function takes the form:

(4) $$P_{\mathrm{acc}}=\exp\left[\frac{-K}{T}\left(\frac{1}{\text{Current tree length}}-\frac{1}{\text{Candidate tree length}}\right)\right]\text{,}$$

**Fig. 5:**
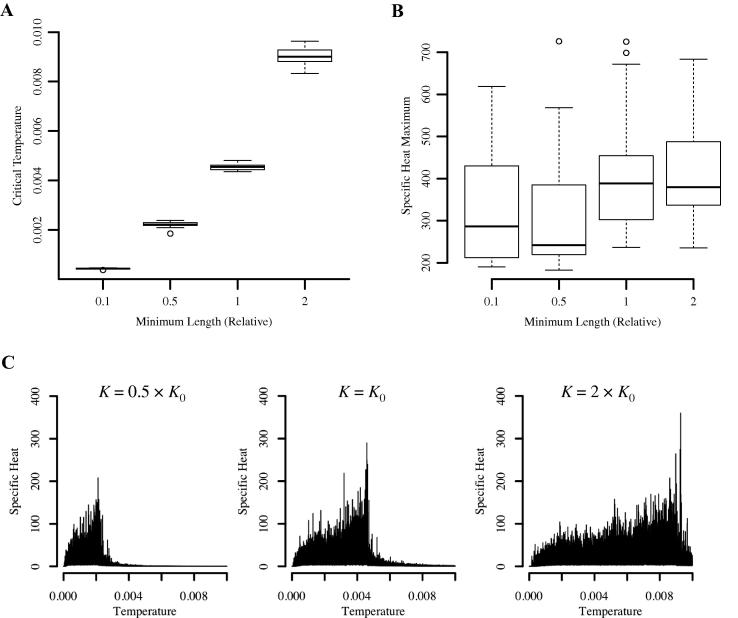
Change in the specific heat profile as the Boltzmann constant is varied. In LVB the minimum length *K* (Eq. (1)) acts as the Boltzmann constant. Whilst keeping all other search parameters constant *K* was varied to 0.1, 0.5 and 2 times its original value, *K*_0_ (20 replicates per value of *K*; original value indicated by ‘1’ in A and B). The results shown here are for ‘Salicini1.smh’. As *K* is increased we see a shift of the maximum to higher temperatures (Anova, *F*_3,76_ = 7335, *p* < 2 × 10^−16^; see A) and an increase in magnitude of the peak (Anova, *F*_3,76_ = 3.409, *p* = 0.02; see B). The three plots in (C) illustrate this. *K* increases from left to right (Left: *K* = 0.5 × *K*_0_, Center: *K* = *K*_0_; Right: *K* = 2 × *K*_0_). With higher values of *K* the peak shifts to higher temperatures and appears prolonged. Furthermore, the magnitude of the peaks increases.

Whilst the value for *K* is in principle determined by the multiple alignment, from a computational point of view it is merely a constant and can be varied. At any given temperature greater values of *K* lead to a stronger tendency towards downwards moves. At the same time the effect of *K* is countered by the temperature, *T*, so that for an increased *K* we will still see identical search space sampling just at higher temperatures. Since the specific heat profiles reflect search space sampling, we therefore expect the peak to shift to higher temperatures if *K* is increased. In addition, changing *K* will change the magnitude of the specific heat peaks since *K* also appears in the expression of the HI of a particular tree (Eq. (1)). An increase in *K* will therefore also lead to an increase in the variance in HI and so specific heat (Eq. (2)). This is precisely what we observe in experiments (Fig. 5; Section S4). When increasing *K* we see a shift of the maximum to higher temperatures and an overall horizontal and vertical stretching of the peak. This illustrates how parameterisation can manifest itself in the specific heat profile.

A useful application of the specific heat profile could be as a diagnostic for setting up the search. We discussed how the search space structure and the value of *K* are reflected in the peak. We propose that, with further research, it might be possible to use the shape and position of the peak to assess the parameterisation and conditioning of the search. As such, the profile could inform on a poorly suited neighbourhood function, too-fast cooling or conflicts in the selected sequences. This would allow one to optimise simulated annealing-based inference algorithms for MP problems and is plausibly also transferable to ML inference and beyond. To illustrate the idea and stimulate future research we conclude with presenting a proof-of-concept application in the next section. We use the specific heat profile as a point of orientation to choose the initial temperature for the search.

### 3.4 Application to the initial temperature problem

A key parameter that has to be set in a simulated annealing implementation is the initial temperature. Too-low values result in suboptimal solutions, whilst too-high starting values waste run time (van Laarhoven and Aarts, 1988, Reeves, 1996).

We benchmarked the performance of LVB starting from three different initial temperatures. One temperature was the critical temperature, identified as the temperature at which the specific heat reaches its maximum. The remaining two were picked at equal distances above and below the critical temperature. To rule out improvements merely due to increased run time at higher temperatures, we fixed the number of iterations across all temperatures for each file. We found that in one of the five alignments tested, starting at the critical temperature yielded the best final results on average (Section S5); in one case, the critical temperature achieved a tree shorter than any other found for this file; in two further cases there was no noticeable differences amongst temperatures; and the lower temperature outperformed the critical temperature search in one case (Section S5). Currently, it is difficult to draw strong conclusions from this benchmarking. Nevertheless, it illustrates how the specific heat profile can be used as a point of orientation for setting search parameters.

### 3.5 Broader relevance to phylogeny reconstruction

Empirical studies of biological accuracy – for example, the ability of a method to retrieve a simulated phylogenetic tree (e.g. Huelsenbeck, 1995, Yoshida and Nei, 2016) – are important, but are not our current topic. For global criteria such as MP, biological accuracy is influenced both by the explicit or implicit model of substitution and by our ability to find the optimal solution according to the criterion. This ability depends on the search algorithm, its implementation and our computer resources. Our focus has been on the simulated annealing search algorithm, with MP as criterion. A different class of methods, such as neighbour joining (Saitou and Nei, 1987), construct a tree algorithmically (Swofford et al., 1996), bypassing the requirement for a search of tree-space. This increases speed, perhaps at the expense of biological accuracy (Huelsenbeck, 1995; cf. Yoshida and Nei, 2016). Further empirical comparisons, including global and algorithmic approaches, will be important as methods continue to develop. Improved heuristic searches will reduce bias and variance in the solution, allowing a more direct empirical assessment of global criteria.

We propose that our study will be applicable to phylogeny reconstruction methods other than MP. Due to conceptual links between MP and ML, we expect broadly similar search-space characteristics in a simulated annealing search for the ML tree (e.g. Salter and Pearl, 2001) and that specific heat profiles could serve for diagnosis also for ML algorithms. Statistically consistent ML phylogeny reconstruction requires an explicit model of substitution, which affects the topology of the final tree (e.g. Hoff et al., 2016). Future studies of the interaction between substitution model and search space may be valuable.

## 4 Conclusion

Thanks to its construction on physical principles, simulated annealing is one of the best-understood heuristics for solving complex optimisation problems. However, many of the theoretical results are of limited help in practice and so ‘rough-and-ready’ practical tricks are required (Ingber, 1993, Reeves, 1996, Ledesma et al., 2008). In what appears to be the largest empirical investigation to date, on 34 real world case studies, we find that specific heat profiles differ significantly between input files and parameterisations. We propose closer study of simulated annealing phase transitions for use in categorising problem cases and as a diagnostic to assess conditioning of the problem and parameterisation of the algorithm. We argue specific heat profiles reflect landscape structure of the problem, which in turn reflects difficulty of the problem instance and setup of the algorithm. Our results have been taken into consideration in new releases of LVB (version 3.0 Beta onwards; http://lvb.bio.ed.ac.uk). Because of the general nature of the simulated annealing approach they are likely to find further application, in phylogeny reconstruction and beyond.

The notion of a particularly effective range of temperature values for simulated annealing is well established and many studies have used this knowledge to obtain higher quality results more rapidly (see for example Sen and Stoffa, 1991, Cohn and Fielding, 1999, Fielding, 2000, Shojaee et al., 2010). However, because of the wide range of areas in which these applications have arisen, the phenomenon is described and studied under a variety of names, including ‘critical temperature’ (Cai and Ma, 2010) or ‘mushy state’ (Shojaee et al., 2010). Although our interpretation in terms of trapping dynamics is physically more analogous to a glass rather than a phase transition (Hasegawa, 2012, Ingber, 1993), we have nevertheless kept the name ‘phase transition’ to link in with perhaps the most popular account of this phenomenon (Kirkpatrick et al., 1983). In addition to a further exploration, creation of a single model and language for the phase transition phenomenon would be a desirable future direction. This could serve as a stepping stone to unite theory and practice in simulated annealing.

## Conflict of interest

We have no competing interests.

## Authors’ contributions

MARS and DB conceived and designed the study, carried out statistical analyses and wrote the manuscript. MARS carried out the computational experiments. Both authors gave final approval for publication.
