# Supplementary material for: On simulated annealing phase transitions in phylogeny reconstruction
Source: Mol Phylogenet Evol. 2016 Aug;101:46–55. doi: 10.1016/j.ympev.2016.05.001 (PMC4912009; doi:10.1016/j.ympev.2016.05.001)
Supplement: Supplementary data 1 [file mmc1.pdf]

On Simulated Annealing Phase Transitions in Phylogeny Reconstruction: Supplementary Data

S1 Alignments and Further Details

The 46 multiple alignments originally assembled from the literature, after processing as outlined in Section 2.2 of the main text, are included in Supplementary Material as a separate zipfile.

A separate spreadsheet in Supplementary Material contains further details for these alignments. Details include the name of the associated paper, first author, number of characters and sequences in the alignment. A short note is provided on how each file was obtained and converted into PHYLIP format. The initial temperatures used for obtaining the specific heat profiles are shown.

S2 Specific Heat Profiles from all 34 Alignments

The main text exemplifies the major types of specific heat profiles observed. To illustrate the full variety of shapes seen in our experiments we here show the profiles for all 34 alignments. Each figure shows a single replicate which was chosen randomly from the 20 replicates performed per alignment (Figures S1–S3). While there is some variation between the replicates, the shape of the curves are well preserved for each input file (see Section 3.1 of the main text). As such, the examples shown are representative of what was observed for all 20 replicates for each alignment. One can see that each input file gives rise to a distinct specific heat profile.

As detailed in section 2.2 of the main text, suitable initial temperatures for each file were found by trying $T_0 = 2 \times 10^{-3}$, 1×10^{-2} or 2×10^{-2}. If no peak was observed for a particular value of T_0 then the subsequent higher

value was tried. Figure S4 shows a specific heat profile for a case where the initial temperature was too low. Instead of a well defined peak only a very variable spike near the beginning of the search is observed.

The peaks differ in three ways. Firstly, variations occur in the direction and amount of skew. While some profiles are nearly symmetric (e.g. "patient6", Figure S2) others are skewed to the left or to the right to varying extents. For example, "1_1399893393_Molecular_noct" (Figure S1) is slightly left skewed while "Pasach_run1" (Figure S2) is right skewed. Secondly, whereas in most cases the progression is smooth, for some instances we observe sudden drops or rises in specific heat as the temperature is decreased (e.g. "Bahl", Figure S1). Lastly, profiles differ in the number of distinguishable peaks. In 29 cases we record a single peak while in 5 cases ("BEAST-MELO-FERREIRArun1", "patient1", "S4", "Ursus_run1_noSpTree", "VATI_ND2_Align_Final") we observe two peaks.

In the case of two peaks there is also great variation in the degree to which the two peaks appear disjoint and their relative magnitudes. We observe instances of complete separation ("VATI_ND2_Align_Final", Figure S3) as well as cases where the peaks are barely disjoint ("S4", Figure S2). In addition, in "Ursus_run1_noSpTree" (Figure S3) the higher temperature peak is more pronounced whereas in "VATI_ND2_Align_Final" (Figure S3) the lower temperature peak is higher. We argue that the heterogeneity in peak shapes can be explained by properties of the particular optimisation problem (alignment) and the paramaterisation of the algorithm.

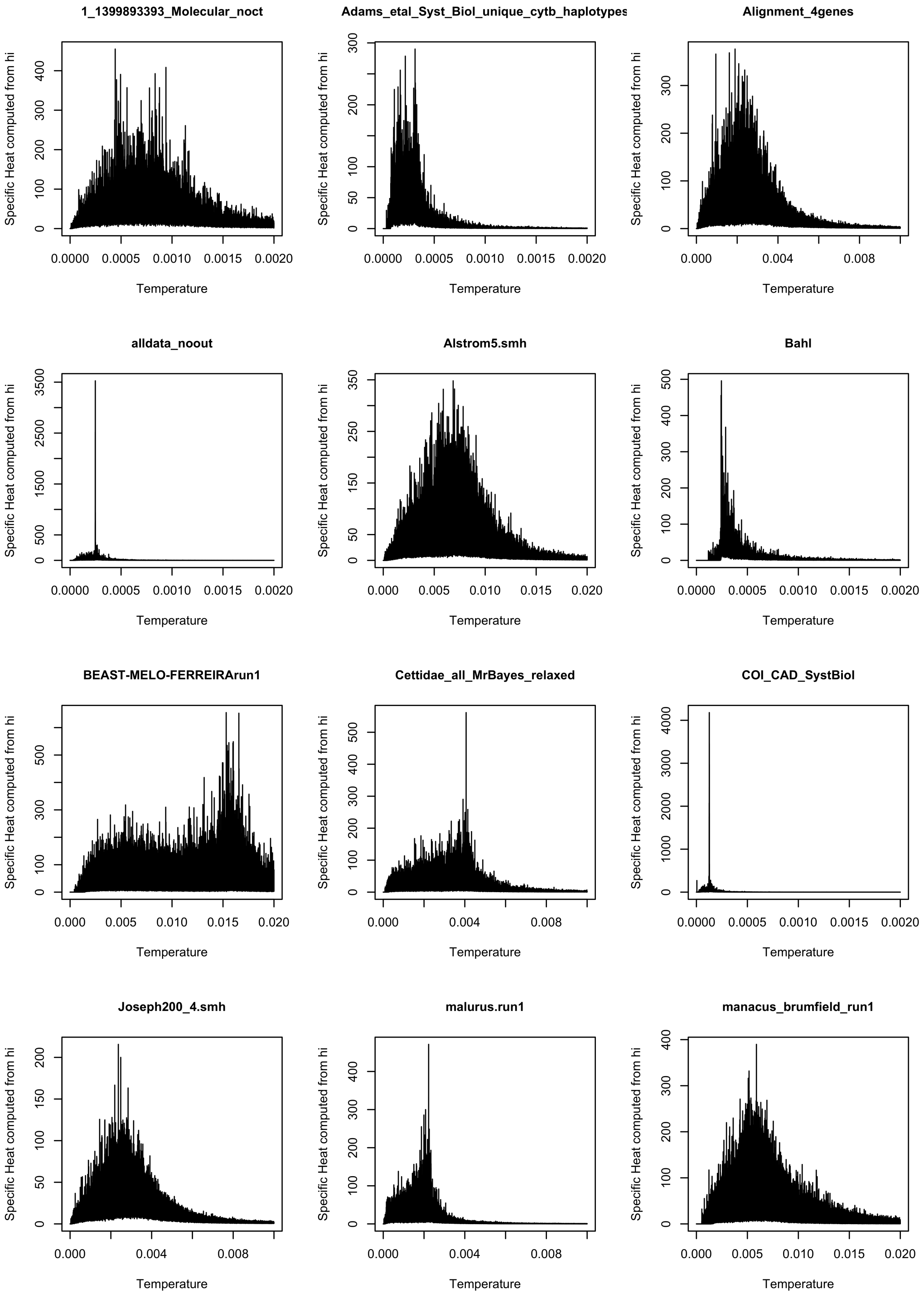

Figure S1: *Exemplifying the various specific heat profiles observed (Alignments "1_1399893393_Molecular_noct" to "manacus_brumfield" in alphabetical order).*

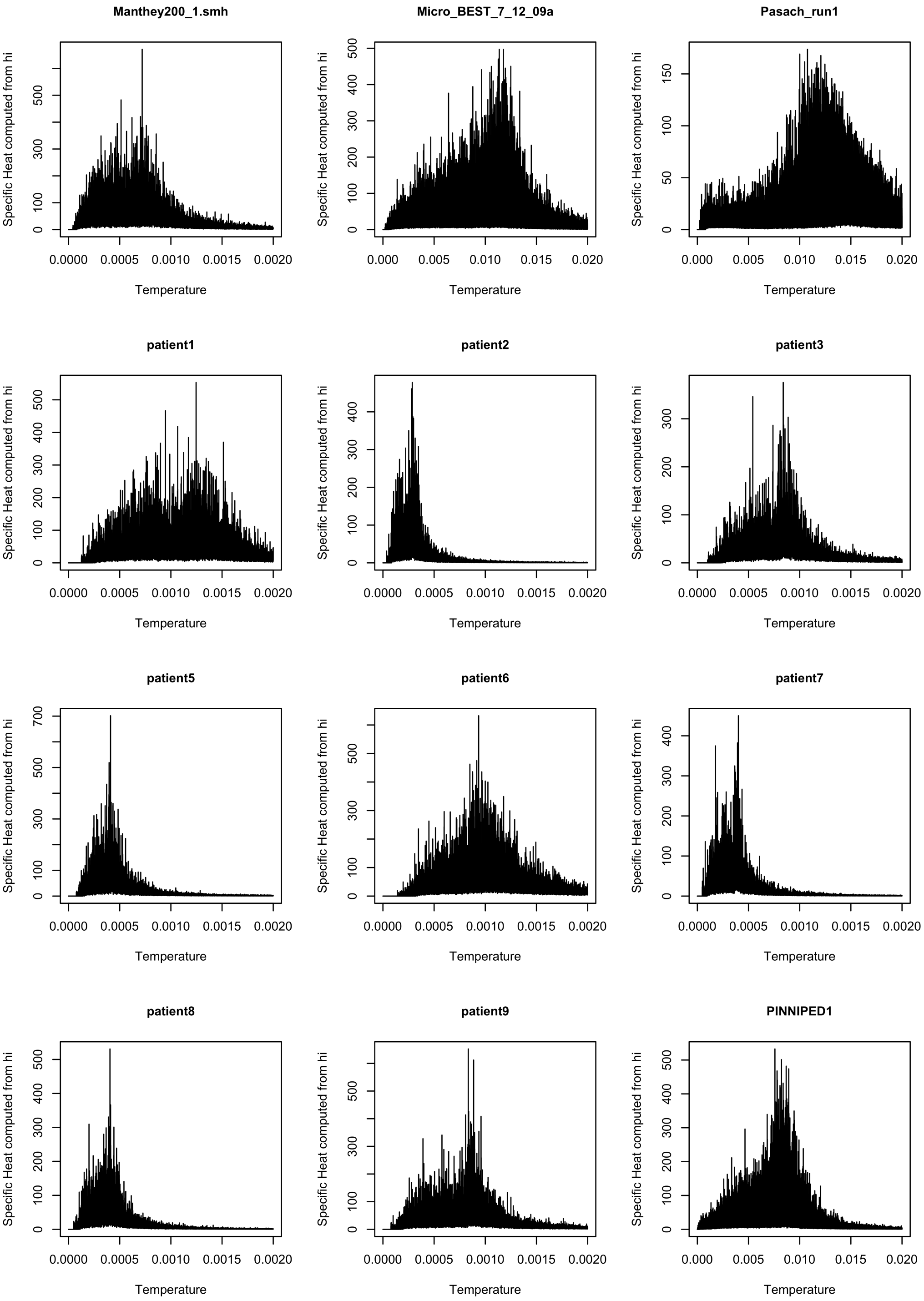

Figure S2: *Exemplifying the various specific heat profiles observed (Alignments “Manthey200_1.smh” to “PINNIPED1” in alphabetical order).*

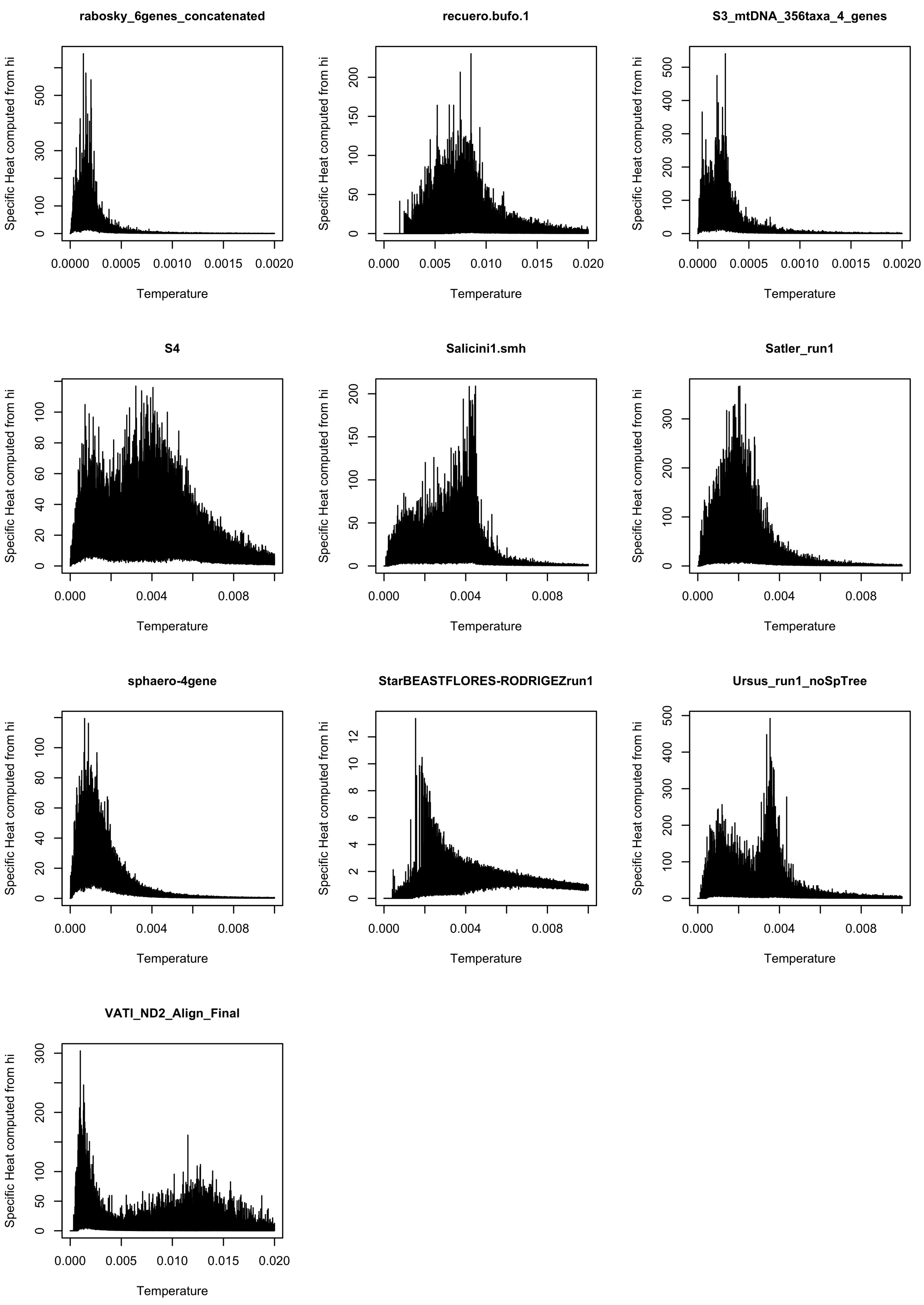

Figure S3: *Exemplifying the various specific heat profiles observed (Alignments "rabosky_6genes_concatenated" to "VATI_ND2_Align_Final" in alphabetical order).*

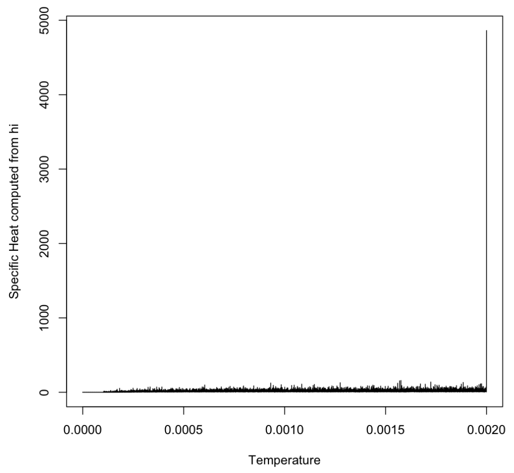

Figure S4: *Showing a specific heat profile when the search commences at a temperature value far below the critical temperature. The alignment used here is "Cettidae_all_MrBayes_relaxed". Instead of a defined peak only a sudden spike in specific heat is observed. The magnitude of the spike varies significantly (see also Figure S13). In these cases a higher initial temperature was tried.*

S3 Visualising Convergence – Multidimensional Scaling Approach

In the main text we visualise convergence of the algorithm by focusing on the structure of the trees currently considered by the algorithm (see section 3.2 and 3.3 in the main text). We show how the trees accepted by our simulated annealing implementation become increasingly similar in structure as the temperature is decreased with a strong non-linearity corresponding to the phase transition. We interpret our observations using a landscape paradigm where the aim of the search is to find the deepest valley in a mountainous area. The phase transition corresponds to the trapping of the search in a particular valley of the search space. We argue that studying the phase transition can give insights into the topography of the search space.

The search space is multidimensional and as such a direct visualisation of the landscape is not possible. However, it is possible to create lower dimensional approximations using the multidimensional scaling technique (Hillis et al., 2005). Trees are represented by points in the plane or in space. To

capture the space's structure, points are arranged such that the Euclidean distance between any two points approximates the topological distance in the tree space between the corresponding trees (Hillis et al., 2005). We used this mutlidimensional scaling technique to attempt to visualise the topography of the search space and link it to aspects of the specific heat profile.

We collected samples of 200 trees accepted by the algorithm at regular points throughout the search on four alignments (in the same manner as described in section 2.3 of the main text; alignments used are "Bahl", "patient3", "patient1", "ursus"; 3 replicates per alignment) and used the Treesetviz package for Mesquite to produce multidimensional scaling plots (Hillis et al., 2005). These plots are plots of points visited by the algorithm where the distance between the points in the plane reflects the unweighted Robinson-Foulds distance between the points in the search space (Hillis et al. 2005). In addition, we coloured the points according to the temperature at which the corresponding tree was collected. Figures S5–S8 show the results for one replicate. The remaining replicates are not shown but look very similar to the ones displayed here. One can see that as the temperature is decreased again the points visited by the algorithm become more similar – the search is restricted to one particular area of the plane (Figures S5B–S8B). While convergence in some cases appears to be smooth (Figure S8B) in other cases the search seems to move from island to island (Figure S12B). This can be interpreted as smoother and rougher landscapes as discussed in Section 3.2 of the main text.

To understand in more detail the role of the phase transition we coloured only the points collected during temperatures of high specific heat – during the phase transition. We find that the points visited during the phase transition encircle the final regions visited (Figures S7–S8). In the case of two transitions we observe two circles. This supports the view that the phase transition correspond to the trapping process.

However, because these are projections of a multidimensional space onto the plane, care has to be taken in the interpretation. For example, only points which have been visited by the algorithm are shown, and as such these are not maps of the search space. In addition, there are potential restrictions in how the points can be arranged on the plane such that not all the visible clustering is necessarily meaningful (For a more in depth discussion see Hillis et al. 2005). Nevertheless, they do represent the search space from the algorithms point of view and approaches like these could be helpful in understanding the structure and challenges of tree search spaces in the future.

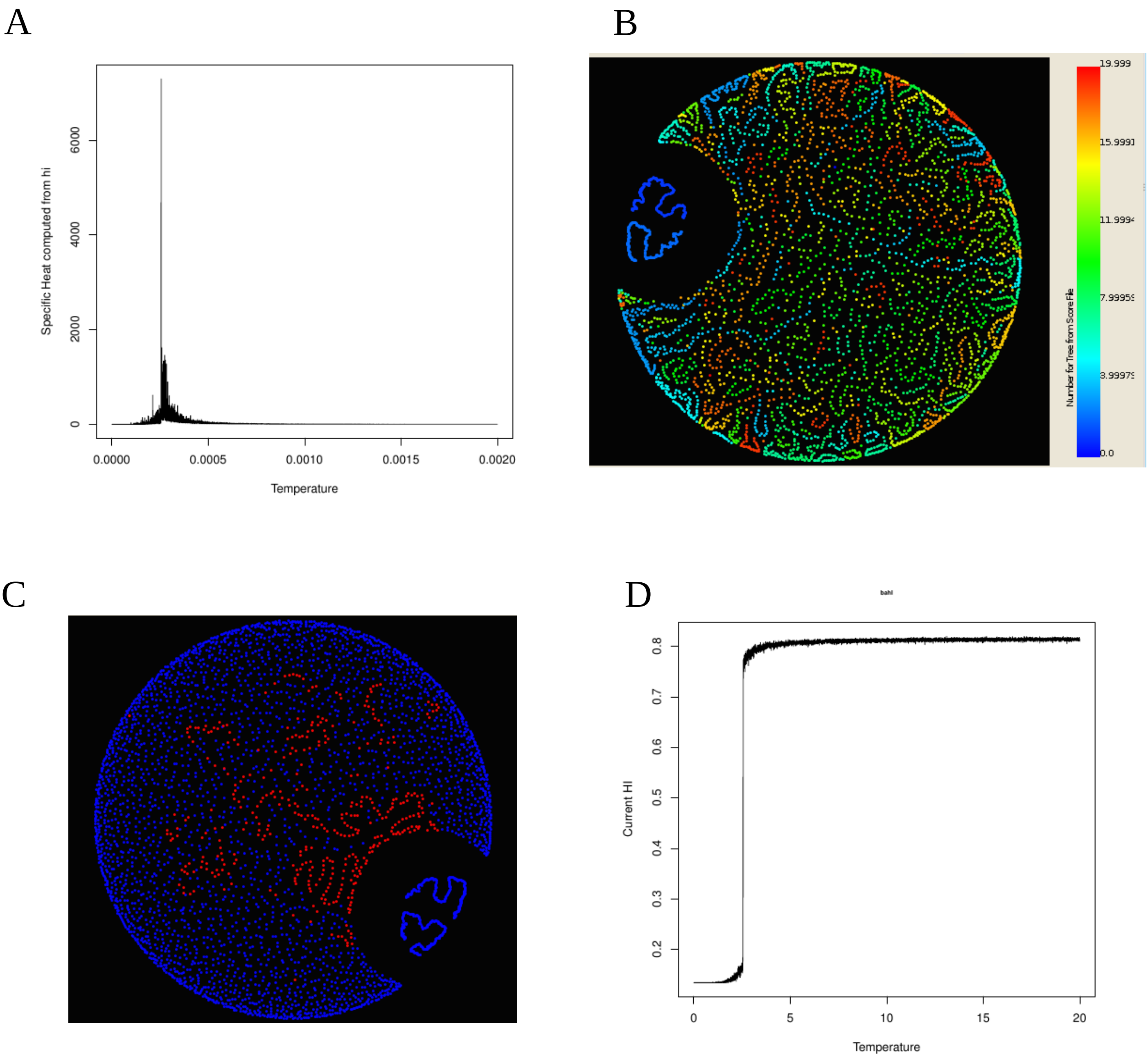

Figure S5: *Convergence of the search for the "Bahl" alignment. (A) shows the specific heat profile and (D) shows the cost of the currently accepted candidate solution. (B) and (C) are multidimensional scalings of samples of 200 accepted trees taken at regular intervals throughout the search. Colouring in (B) is according to temperature at which the tree was collected (red high – blue low; in units 10^{-4}). The convergence to a particular area (island) of the search space is clearly visible. In (C) the trees collected during the phase transition are coloured in red. The observed pattern of "intertangled strings" is very different to the pattern observed for the other files as is the shape of the specific heat profile. The string-like pattern indicates high correlation between successive candidate solutions at nearly all temperatures. We hypothesise the landscape is dominated by a single minimum drawing the search towards it at all times – an "easy" problem.*

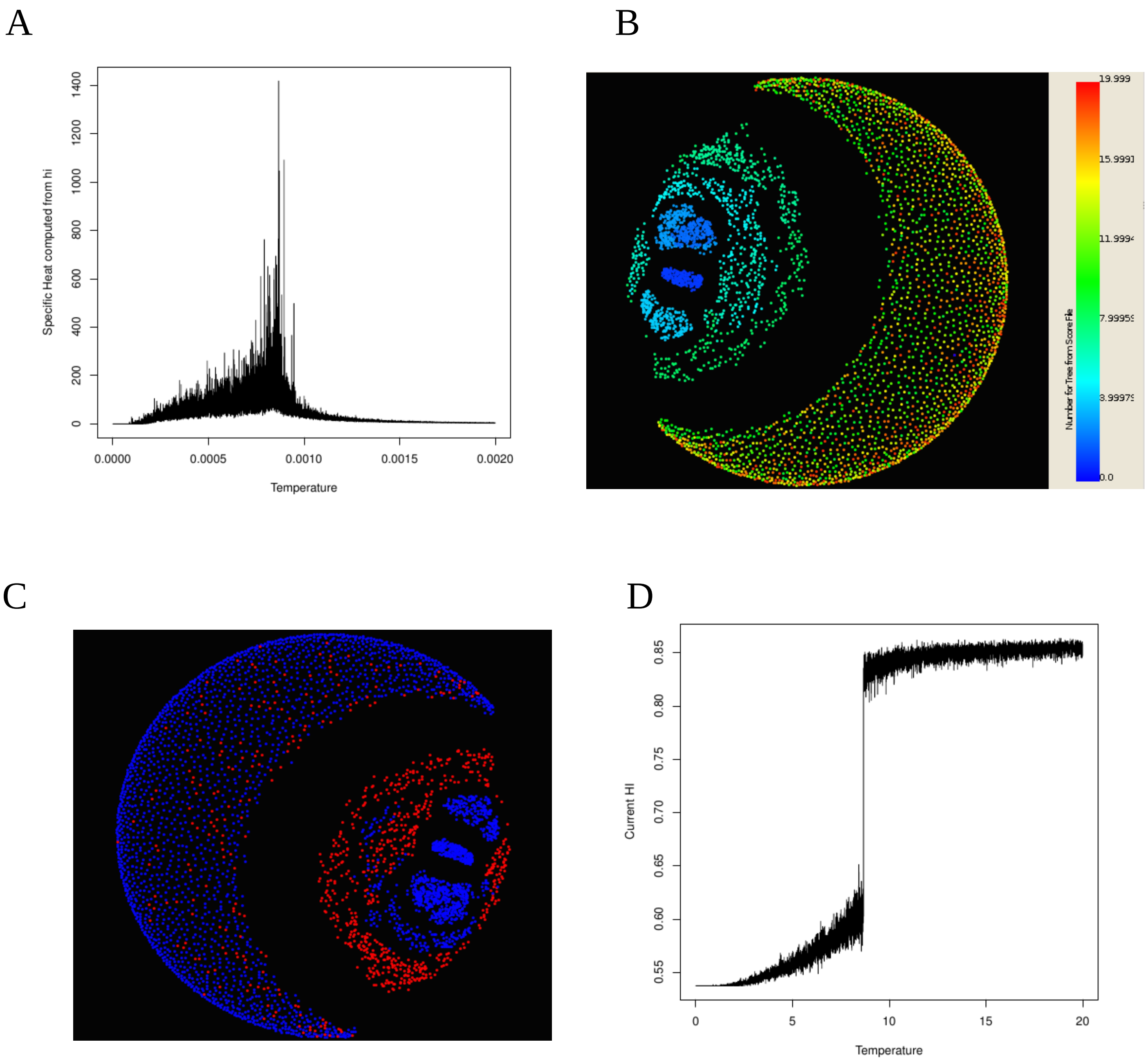

Figure S6: *Convergence of the search for "patient3" alignment. (A) shows the specific heat profile and (D) shows the cost of the currently accepted candidate solution. (B) and (C) are multidimensional scalings of samples of 200 accepted trees taken at regular intervals throughout the search. Colouring in (B) is according to temperature at which the tree was collected (red high – blue low; in units* 10^{-4}*). The convergence to a particular area (island) of the search space is clearly visible. In (C) the trees collected during the phase transition are coloured in red. The trees considered during the period of the phase transition join the outer region and the final island, indicating that the transition coincides with a descent into a particular neighbourhood. The spotted appearance of the final region suggests that there are perhaps several minima present in this neighbourhood – a valley with substructure.*

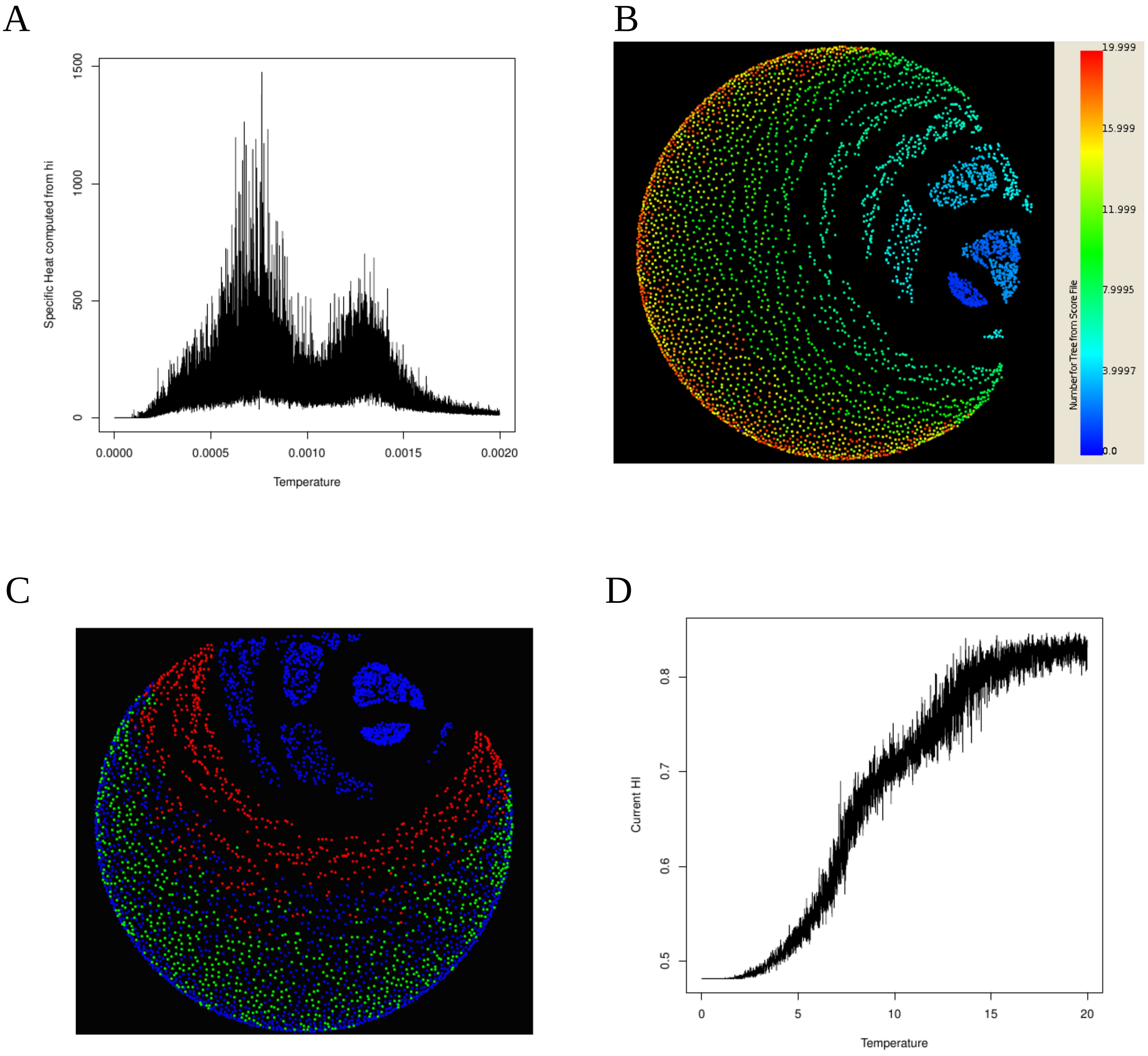

Figure S7: *Convergence of the search for "patient1" alignment. (A) shows the specific heat profile and (D) shows the cost of the currently accepted candidate solution. To obtain better resolution of the two peaks the search here was run for* $n = 20000$ *moves at each temperature. All other search parameters were kept equal. (B) and (C) are multidimensional scalings of samples of 200 accepted trees taken at regular intervals throughout the search. Colouring in (B) is according to temperature at which the tree was collected (red high – blue low; in units* 10^{-4}*). The convergence to a particular area (island) of the search space is clearly visible. In (C) the trees collected during the higher temperature peak are coloured in green. Trees collected during the lower temperature peak are coloured in red. The trees considered during the two peaks form two distinct rings around the area with the final trees. The effect of the higher temperature transition is not very clearly visible, while the lower temperature transition marks a descent into a confined region, again with some substructure.*

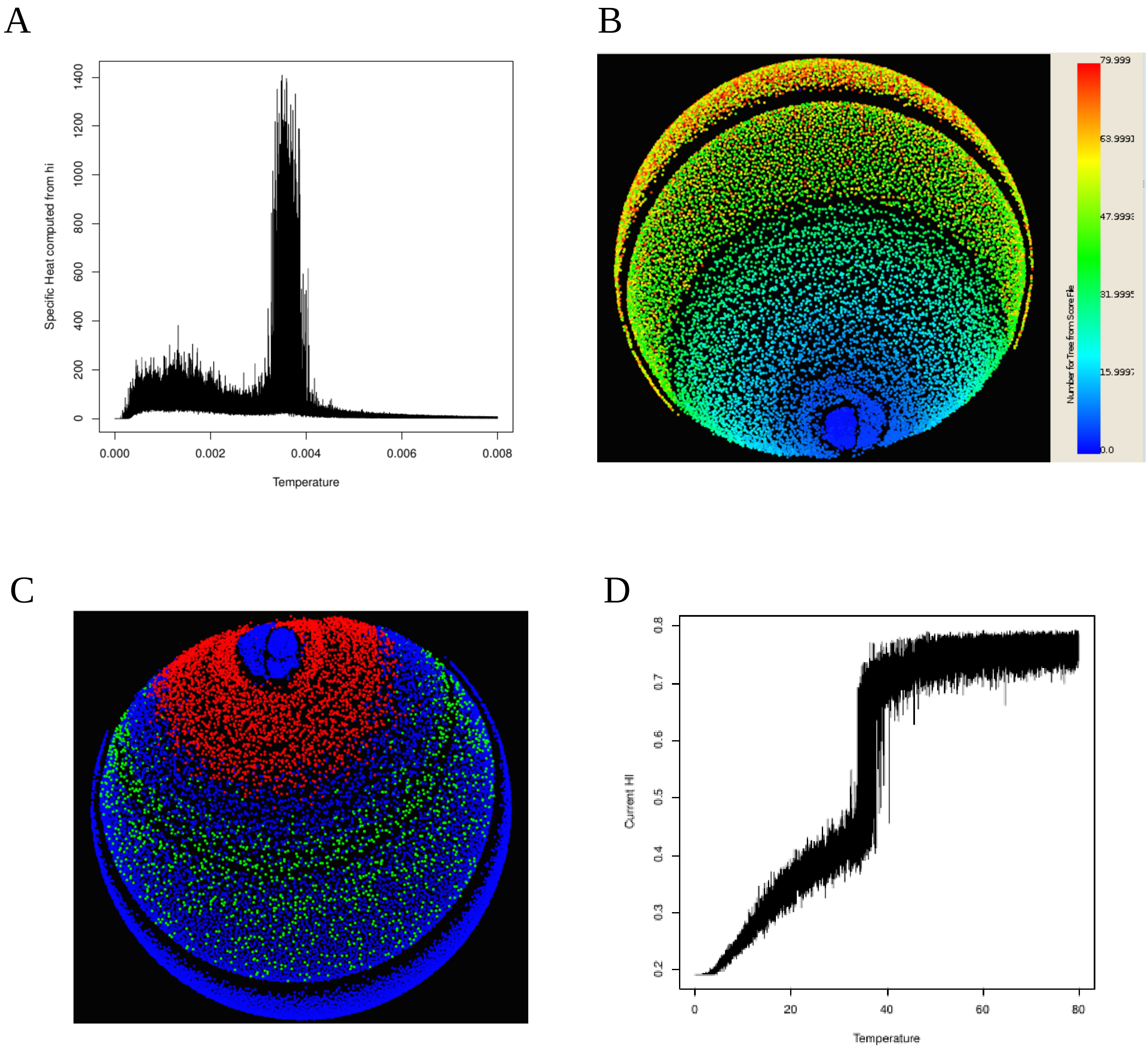

Figure S8: *Convergence of the search for "ursus" alignment. (A) shows the specific heat profile and (D) shows the cost of the currently accepted candidate solution. To obtain better resolution of the two peaks the search here was run for $n = 200000$ moves at each temperature. All other search parameters were kept equal. (B) and (C) are multidimensional scalings of samples of 200 accepted trees taken at regular intervals throughout the search. Colouring in (B) is according to temperature at which the tree was collected (red high – blue low; in units 10^{-4}). The convergence to a particular area (island) of the search space is clearly visible. In (C) the trees collected during the higher temperature peak are coloured in green. Trees collected during the lower temperature peak are coloured in red. Again, the trees considered during the two peaks form two distinct rings around the area with the final trees. Unlike in the previous cases, after an initial jump the pattern of convergence appears almost smooth. We hypothesise that clusters of distinct minima compete to attract the search, during the first transition the cluster is set (trees still comparably different), during the second the particular minimum (trees very similar, but island structure for different minima).*

S4 Varying the Minimum Length, *K*

To illustrate how changes in a search parameter affect the characteristics of the specific heat curves we varied the minimum length parameter K to 0.1, 0.5, 2 and 10 times its original value for 5 multiple alignments (See section 2.3 of the main text). For each value of K we performed 20 replicates. In the following we present the results for the files not shown in the main text (Figures S9–S12). In all but one case we see the same pattern as outlined in the main text. For the "Bahl" alignment we curiously see a decrease rather than an increase and hypothesise this is linked to the unusual shape of the profile (Figure S11).

The results for 10 times the original K are not displayed as the peaks in specific heat had moved out of the range of temperature values considered. This manifested itself as shown in Figure S13 for the "Salicini1.smh" alignment. The specific heat curves were very unstable giving rise to sudden spikes in specific heat near the beginning of the search rather than the well defined curves. As a result we excluded the 10 times case from further analysis.

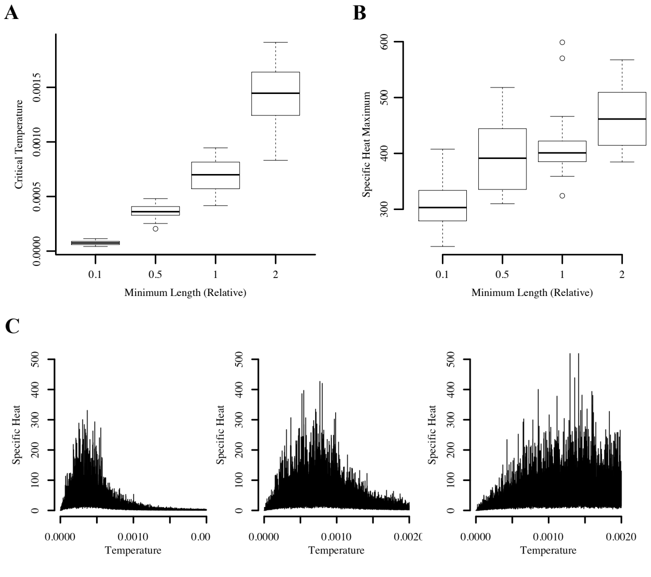

Figure S9: *Varying K when searching for the optimal tree for the "1_1399893393_Molecular_noct" alignment. As K is increased we see (A) a shift of the maximum to higher temperatures and (B) an increase in magnitude of the peak. The three plots in (C) illustrate this further. K increases from left to right (Left: K = 0.5 × original value, Center: K = original value; Right: K = 2 × original value).*

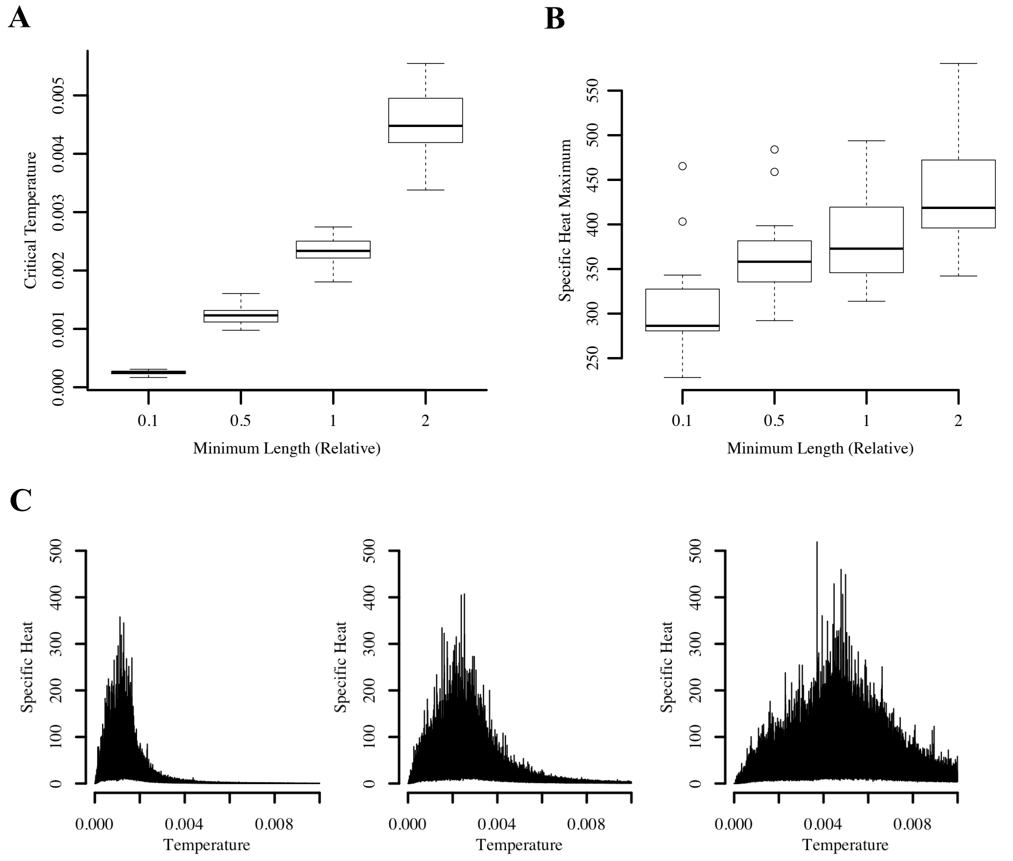

Figure S10: *Varying K when searching for the optimal tree for the "Alignment_4genes" alignment. As K is increased we see (A) a shift of the maximum to higher temperatures and (B) an increase in magnitude of the peak. The three plots in (C) illustrate this further. K increases from left to right (Left: K = 0.5 × original value, Center: K = original value; Right: K = 2 × original value).*

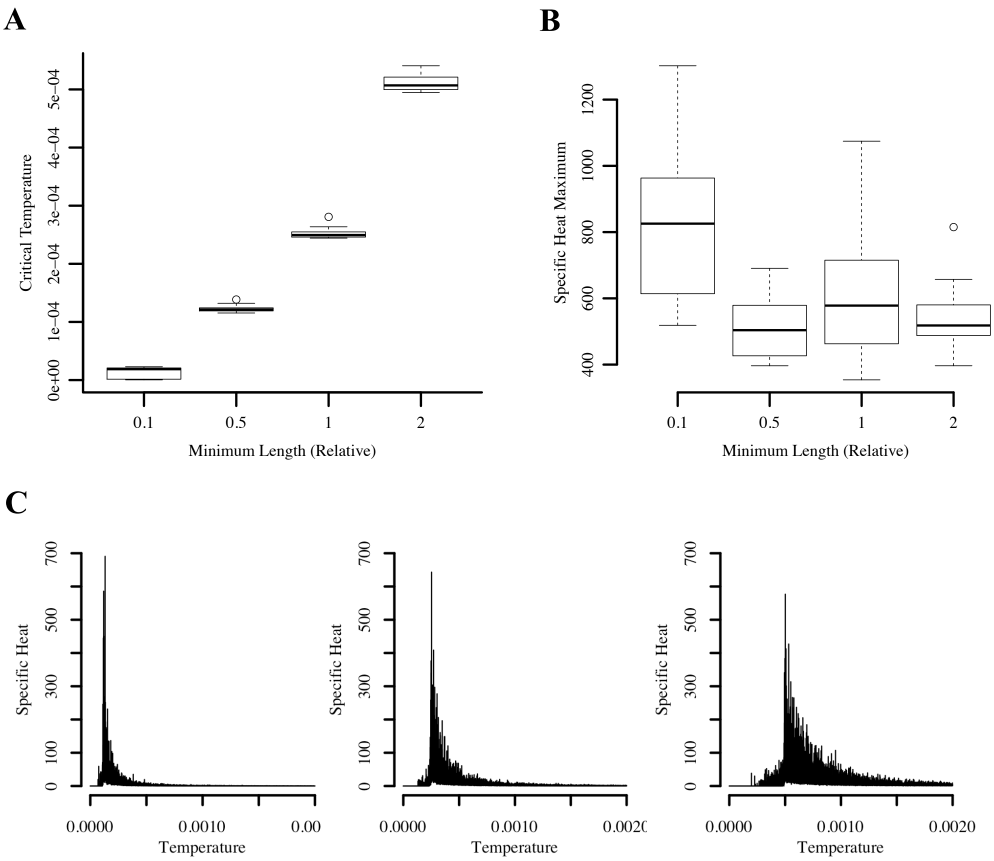

Figure S11: *Varying K when searching for the optimal tree for the "Bahl" alignment. As K is increased we see a shift of the maximum to higher temperatures (See A) and interestingly a decrease in magnitude of the peak (See B). The three plots in (C) illustrate this further. K increases from left to right (Left:* $K = 0.5 \times$ *original value, Center:* $K =$ *original value; Right:* $K = 2 \times$ *original value). The decrease in magnitude is in contrast to the increase found in the other cases. The underlying mechanisms are still to be investigated but it seems plausible that this is linked to the same characteristic of the alignment which is also responsible for the unique spike shape of the peak.*

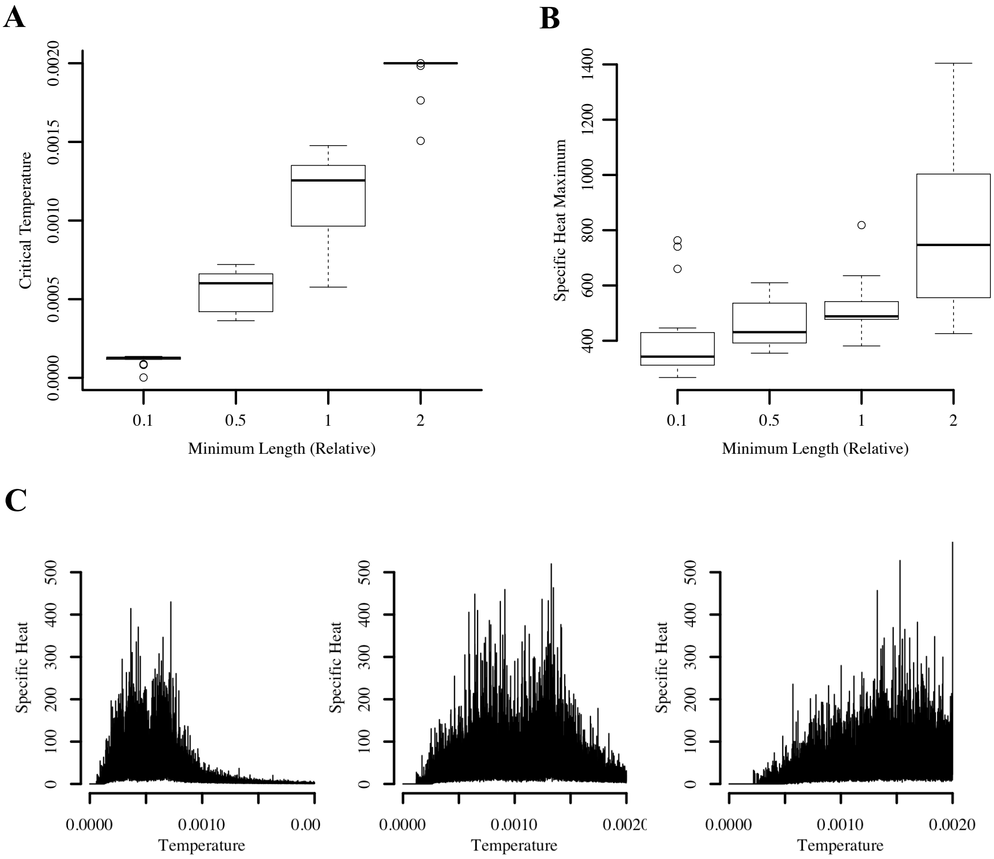

Figure S12: *Varying K when searching for the optimal tree for the "patient1" alignment. As K is increased we see (A) a shift of the maximum to higher temperatures and (B) an increase in magnitude of the peak. The three plots in (C) illustrate this further. K increases from left to right (Left: K = 0.5 × original value, Center: K = original value; Right: K = 2 × original value).*

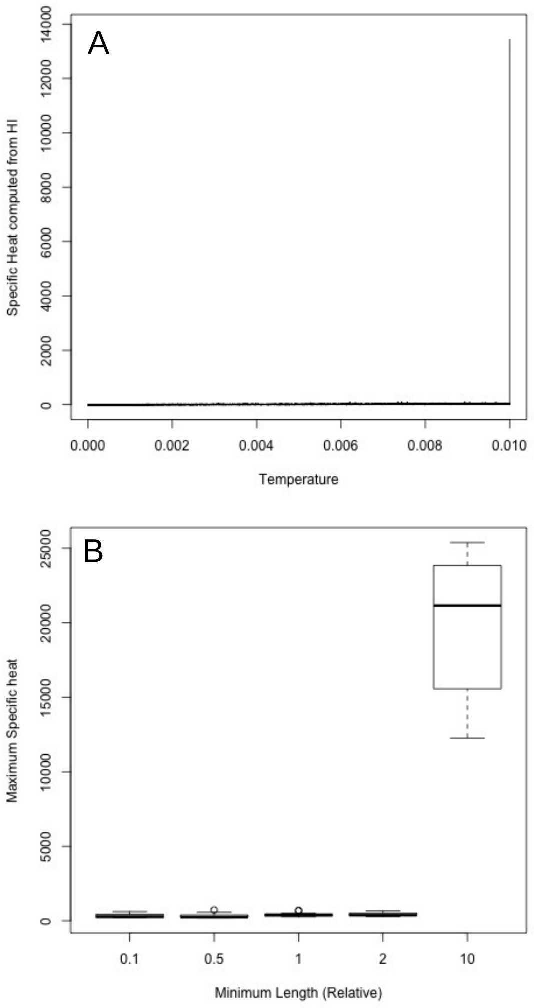

Figure S13: *Exemplifying the specific heat curves obtained when setting K to 10 times its original value (Here for one replicate on "Salicini1.smh"). (A) No well-defined peak is visible but instead the specific heat is very large at the first temperature value of the search and then drops dramatically. (B) Magnitude of this spike is highly variable. This is because the critical temperature is now well above the temperature range considered ([10^{-8},10^{-2}]; compare with Figure S4). Because of the high variability, and since no peak can be seen, the 10 times case was not considered further.*

S5 Application to the Initial Temperature Problem

To illustrate how one might use the specific heat profile to improve the algorithm we utilised the specific heat profiles to choose suitable values for the initial temperature parameter. We compared searches starting at the critical temperature for each file (“T_2”) with searches starting at a lower (“T_1”) and a higher temperature (“T_3”). The detailed proceedings can be found in Section 2.4 of the main text. Table S1 gives the initial temperatures and the number of rearrangements for each benchmarking file.

Figure S14 shows the result of the benchmarking experiments. In one case the search starting from the critical temperature, T_2, consistently outperforms the other searches (“Alignment_4genes”; Anova, $F_{2,57} = 24.15$, $p = 2.53 \times 10^{-8}$). For one further benchmarking case, T_2 is outperformed on average by the T_1 search (Anova, $F_{2,57} = 8.43$, $p = 6.21 \times 10^{-4}$) but finds finds a solution exceeding all found by any other search (“Bahl”). For “patient3” and “Salicini1.smh” there is no statistically significant difference (Anova, $F_{2,57} = 2.028$, $p = 0.141$ and $F_{2,57} = 1$, $p = 0.374$). In the search on the “1_1399893393_Molecular_noct” alignment the critical temperature search, T_2, is outperformed by the T_1 search (Anova, $F_{2,57} = 60.75$, $p = 7.43 \times 10^{-15}$). This shows that by starting at the critical temperature better results can be achieved, however, further research will be required to fully utilise the specific heat profile.

File	T_1	T_2	T_3	Number of Rearrangements
1_1399893393_Molecular_noct	0.0001	0.000743465	0.00138693	397564
Alignement_4genes	0.0001	0.00221848	0.00433696	696625
Bahl	0.0001	0.00025389	0.00040778	223854
Patient3	0.0001	0.0008492	0.0015984	433953
Salicini1.smh	0.0001	0.00450514	0.00891028	827415

Table S1: Initial temperatures and number of rearrangements for each benchmarking file.

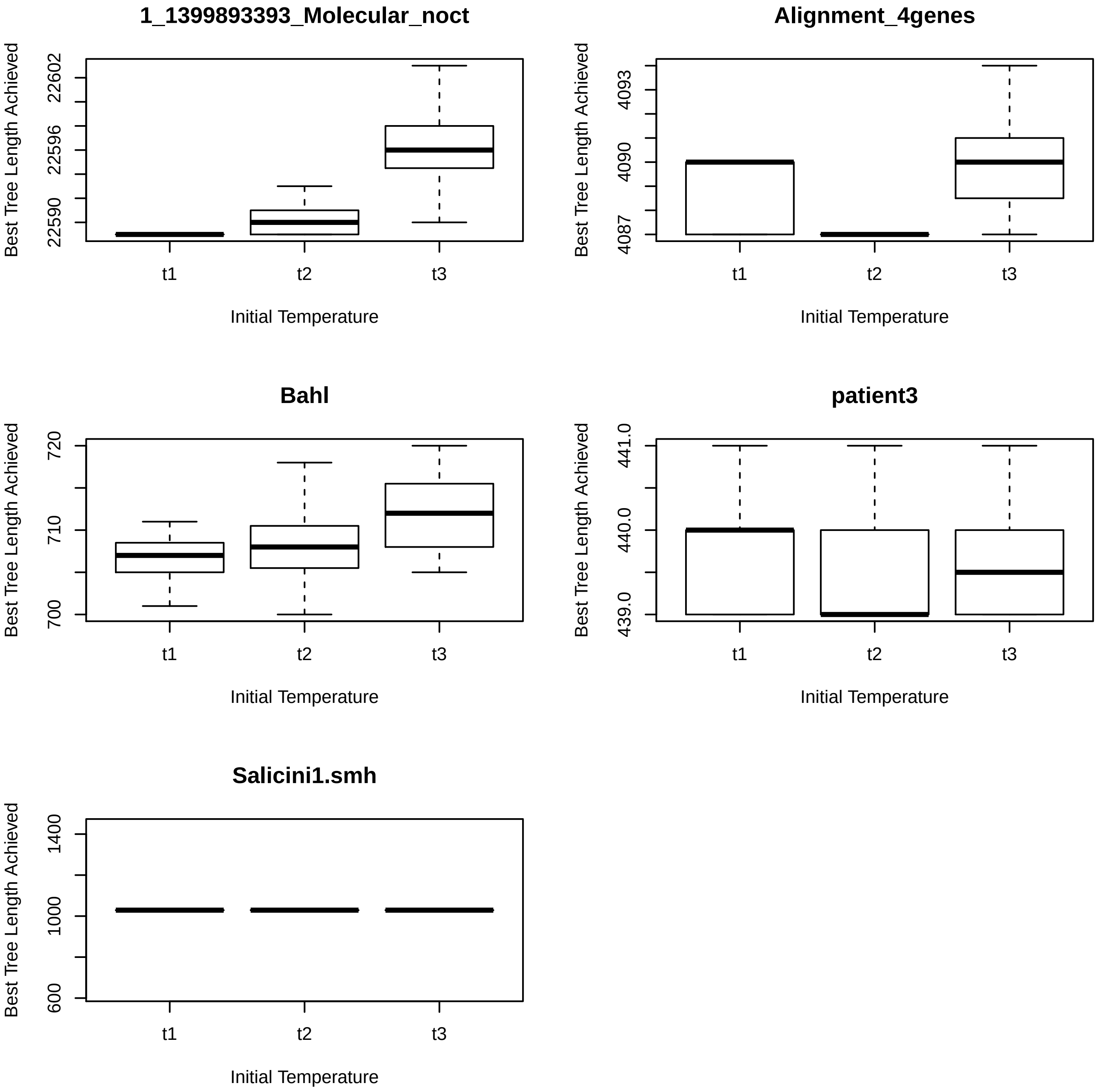

Figure S14: *Showing the benchmarking results when starting the search from different initial temperatures.* T_2 *was chosen using the specific heat profiles in Figures S1–S3. This application illustrates how the profiles might be used but also indicates that further research will be required to better understand and utilise them.*

References

[1] Hillis D. M. et al. (2005). *Analysis and Visualization of Tree Spaces.* Syst. Biol. 54(3):471-481.
